# Ultra-broadband single-stack mid-infrared semiconductor lasers grown by MOCVD

**DOI:** 10.1038/s41377-026-02268-8

**Published:** 2026-04-10

**Authors:** Peng Liu, Lequan Zhang, Yujin Wu, Huanyu Lu, Sicong Tian, Bo Meng, Qi Jie Wang, Cunzhu Tong, Lijun Wang

**Affiliations:** 1 State Key Laboratory of Luminescence Science and Technology, Changchun Institute of Optics, Fine Mechanics and Physics, Chinese Academy of Sciences, Changchun, China; 2 University of Chinese Academy of Sciences, Beijing, China; 3 School of Electrical and Electronic Engineering, Nanyang Technological University, Singapore, Singapore; 4 Bimberg Chinese-German Center for Green Photonics, Changchun Institute of Optics, Fine Mechanics and Physics, Chinese Academy of Sciences, Changchun, China

**Keywords:** Quantum cascade lasers, Semiconductor lasers

## Abstract

Quantum cascade lasers are highly desirable for chemical, physical, and biological research scenarios. Among the applications, mid-infrared frequency combs based on quantum cascade lasers have sparked increasing interest due to their unique advantages in small footprint, high power, and flexible designability. Despite significant performance improvement over a decade, the lasing spectrum bandwidths of the quantum cascade laser frequency combs are still limited to ~100 cm^−1^, limiting their applications in multi-gas spectroscopy and posing severe challenges in tracking their carrier-envelope offset frequency. To achieve a broad-gain spectrum, heterogeneous active regions consisting of multiple stacks of different wavelengths have been implemented. For example, stacking active regions of four different wavelengths results in a full width at half maximum of approximately 0.92 μm (110 cm^−1^) at 290 K, and of ~2.7 μm (360 cm^−1^) at 80 K. However, as more stages are stacked, ensuring a homogeneous and flat gain profile from both design and growth perspectives becomes very challenging. In this work, we present a demonstration of ultra-broadband quantum cascade lasers with a diagonal multi-state-to-continuum active region design. The proposed active region design exhibits a surprisingly wide electroluminescence with a full width at half maximum of ~600 cm^−1^ at 298 K. Devices, with a total peak output power of 2.72 W and a slope efficiency of 1.3 W/A, have shown a lasing spectrum of ~1 μm over 43% of the current dynamic range, with a maximum bandwidth of 1.2 μm around the rollover current. Moreover, a much broader lasing bandwidth of 1.93 μm is obtained from the same device at 80 K, accounting for 22% of the center wavelength. This work represents substantial progress on the single-stack ultra-broadband mid-infrared semiconductor lasers and may provide a novel platform for mid-infrared frequency combs, which are of paramount importance to broadband high-precision spectroscopy, imaging, and free-space communication systems.

## Introduction

Since their initial demonstration in 1994^1^, quantum cascade lasers (QCLs) have undergone continuous and substantial performance improvements, driven by advances in laser design, material growth techniques, and packaging technologies^2,3^. A broadband gain QCL is essential for various laser applications, such as multi-gas spectroscopy^4–7^, medical diagnostics^8–10^, and ultrashort pulse generation^11–13^. It is also crucial for QCL-based frequency combs driven by the cavity nonlinearity. Recent progress has witnessed a boost in the QCL frequency comb output power. However, the long pursuit ‘self-referencing’ *f–*2 *f* method^14^, a technique to fully stabilize a comb and that requires an octave-spanning emission spectrum, has not been demonstrated due to limited gain bandwidth. To achieve a broad-gain profile, stacking QC cores with different center peaks is the most straightforward method^15^. Although this design effectively broadens the gain bandwidth, it also increases thermal resistance and threshold current, as the limited number of cascade stages in each sub-stack results in insufficient peak gain, thereby hindering continuous-wave (CW) operation. Furthermore, strong mode competition in the multi-stack structure exacerbates the spatial hole-burning effect, and thus induces spectral gaps^16^. Although some active region designs can yield a broad-gain profile using a single QC stack, such as bound-to-continuum (BTC)^17^ and dual-upper-state (DAU)^18,19^, further bandwidth enhancement to 600 cm^−1^ remains absent in the 8–10 μm wavelength range. Besides active region design, material growth technology has also seen significant developments. Compared with molecular beam epitaxy (MBE), which has been predominantly used for earlier broadband QCL developments, metal organic chemical vapor deposition (MOCVD) has proven to be a strong competitor for high-quality material growth due to the advantages of high efficiency, short maintenance cycles, and high stability^20–27^. Therefore, single-stack ultra-broadband QCLs grown by MOCVD bear great importance for both fundamental research and industry applications.

In this work, we present an ultra-broadband single-stack QCL design based on a diagonal multi-state-to-continuum (MTC) design which exhibits a bias-insensitive electroluminescence (EL) spectrum with a full width at half maximum (FWHM) of ~600 cm^−1^ at a center wavelength of 8.8 μm. The device with the proposed active region demonstrates a lasing spectrum bandwidth >1 μm over 43% of the current dynamic range, with a maximum bandwidth of 1.2 μm. Despite the broad-gain spectrum, high laser performance was demonstrated with a total peak output power of 2.72 W and a slope efficiency of 1.3 W/A at 298 K. Moreover, a lasing bandwidth of 1.93 μm is demonstrated from the same device at 80 K. Compared with other broadband designs, the MTC design not only achieves the broadest EL and laser spectra, but also exhibits competitive performance in maximum output power, slope efficiency, and wall-plug efficiency (WPE), enabling a broad laser spectrum while maintaining excellent overall performance. Moreover, mode competition effects have been observed in the MTC device incorporating a single-stack active region within a narrow cavity, and a transverse mode competition model based on the iterative rate-equation method has been established, showing good agreement with the experimental results.

## Results

### Active region design

**Fig. 1:**
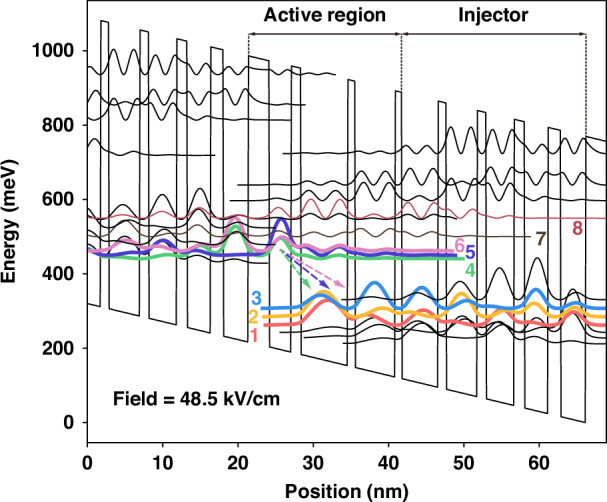
Conduction band diagram and moduli squared of the relevant wavefunctions of the diagonal multi-state-to-continuum active region design under an applied electric field of 48.5 kV/cm. The layer sequence of one period (in nanometers), starting from the injection barrier, is as follows: **2.78**/2.98/**1.24**/6.24/**0.99**/5.32/**0.87/**4.82/**1.08**/4.14/**1.21**/3.64/**1.41**/3.13/**1.67**/3.28, where InAlAs barriers are in bold, and the underlined numbers correspond to the doped layers (Si, 1.5 × 10^17^ cm^−3^)

To broaden the gain spectrum, the fundamental principle of bandstructure engineering is to utilize multiple upper and lower laser levels in order to facilitate multiple laser transitions. To ensure a flat gain profile over a large bandwidth, the oscillator strengths of all transitions should be engineered to be evenly distributed. Furthermore, strong coupling among all upper laser levels is crucial to maintain similar electron distributions. Additionally, diagonal transitions are preferable because of the broader bandwidth compared to the vertical counterparts^28–30^. Regarding the material system, enhancing the performance of QCLs requires effectively reducing the electron escape rate from the upper states into the continuum states^26,31,32^. In this context, strain-compensated materials offer distinct advantages over lattice-matched materials due to the larger conduction band offset^33–35^. Taking all these factors into consideration, the strain-balanced In_0.593_Ga_0.407_As/In_0.362_Al_0.638_As material system was used and the proposed conduction band diagram of the MTC design is illustrated in Fig. 1. To enable multiple diagonal transitions across a broad-gain spectrum, three concurrent design rules are adopted: (i) widen the downstream well adjacent to the injection barrier to promote diagonal transition, (ii) shrink the injection barrier to strengthen the coupling among the manifold of injector states, (iii) retain a chirped-injector profile that forces the subbands into a dense, closely spaced energy ladder. Optimizing these conditions, several diagonal transitions with comparable oscillator strengths could emerge. By optimization, two injector states are strongly coupled with the upper laser state and spread into the active region, with an energy splitting of over 20 meV between states 4 and 6. Transitions from all three states could contribute to the gain spectrum. Electrons injected from the injector via resonant tunneling are quickly redistributed almost equally among the upper states due to the strong couplings. In addition to broadening the gain spectrum, the strong coupling also increases the injection efficiency from the injection states into the upper state, reducing the escape rate of the electrons into the parasitic channels. The dipole matrix elements from the upper states to the lower states are designed to share similar values, e.g., z_6,3_ ~ z_5,3_ ~ z_4,3_ ~ 1 nm, ensuring a homogeneously broadened gain spectrum. Meanwhile, the upper triplet and lower states feature a diagonal design to broaden the gain bandwidth and increase the population inversion. The spacing between state 4 and high-energy parasitic state 7 is designed to be large, over 60 meV, to reduce the parasitic effect. However, the strong coupling of the triplet makes the energy separation E_76_ relatively small. Since E_76_ ~ 42 meV is close to the LO-phonon energy ~33 meV of InGaAs^36^, electrons at level 7 are effectively scattered into level 6, thus causing little effect on the injection efficiency into the upper triplet^37^.

The whole QC structure was grown by the MOCVD on an *n*-doped InP substrate (~2 × 10^18 ^cm^−3^), starting with a 0.5 μm thick *n*-doped (5 × 10^16^ cm^−3^) InP layer, followed by a 3.0 μm thick *n*-doped (2 × 10^16 ^cm^−3^) InP cladding layer, the active region (50 stages of strained In_0.593_Ga_0.407_As/In_0.362_Al_0.638_As layers), and a top waveguide cladding [consisting of a 3.3 μm thick *n*-doped (2 × 10^16 ^cm^−3^) InP layer, a 0.4 μm thick *n*-doped (2.5 × 10^17 ^cm^−3^) InP layer, and a thin 0.3 μm thick *n*-doped (2.5 × 10^18 ^cm^−3^) contact layer]. A reference structure with a close transition energy based on the BTC design was also grown by the same growth facility for the gain bandwidth comparison.

### Material growth and characterization

**Fig. 2:**
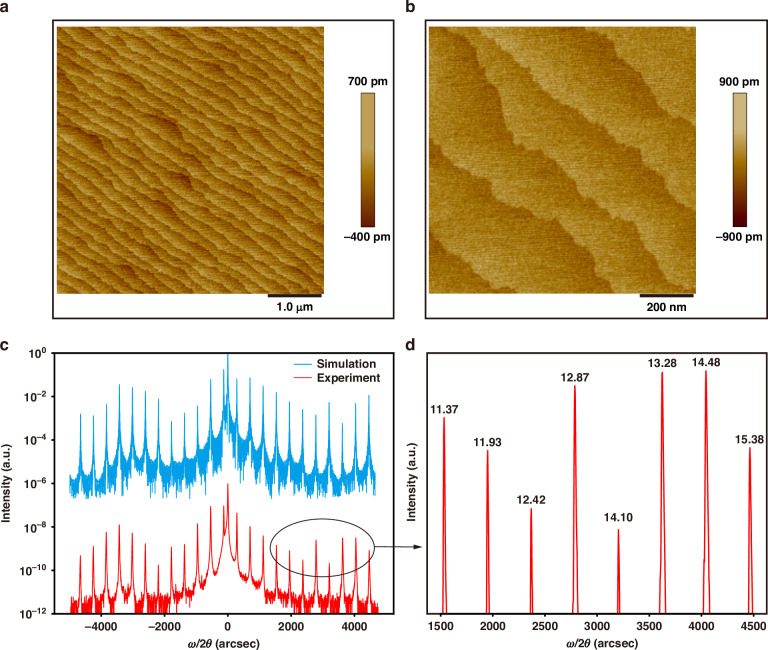
Material characterization of the strain-balanced In_0.593_Ga_0.407_As/In_0.362_Al_0.638_As heterostructure. Atomic force microscopy images of **a** 5 × 5 μm^2^ scan and **b** 1 × 1 μm^2^ scan of a 50-period active region structure. **c** High-resolution x-ray diffraction scan of experimental (red, lower curve) and simulated (blue, upper curve) results of the designed structures. **d** Partially enlarged view of satellite diffraction peaks. The full-widths at half-maximum of satellite diffraction peaks are labeled in arcsec

The surface morphology was examined on the atomic scale by using atomic force microscopy (AFM). Figure 2a shows the surface image of the strained active region measured by AFM in the contact mode (5 μm × 5 μm scan), showing a typical step-flow growth mode feature with kink spacing of ~200 nm. The finer AFM mapping (1 μm × 1 μm scan) in Fig. 2b reveals a calculated surface root-mean-square of 0.16 nm after growing the thick active region.

The periodicity and material quality were further investigated by the high-resolution X-ray diffraction. Figure 2c shows a typical ω/2θ pattern and the corresponding simulation of the whole 50-stage QCL structure employed in this study. The experimental result shows excellent agreement with the simulation, demonstrating excellent control over the key design parameters such as layer thickness, alloy composition, and interface quality. Figure 2d shows the enlarged view of satellite diffraction peaks with FWHM labeled, which are given by fitting the peaks with a Gaussian distribution. A multitude of high-order satellite peaks are distinctly visible, characterized by their extremely narrow FWHM of ~14 arcsec. This small value of FWHM indicates the perfect periodicity and uniformity of the entire structure and the atomic-level roughness of the heterointerfaces.

### Device performance

**Fig. 3:**
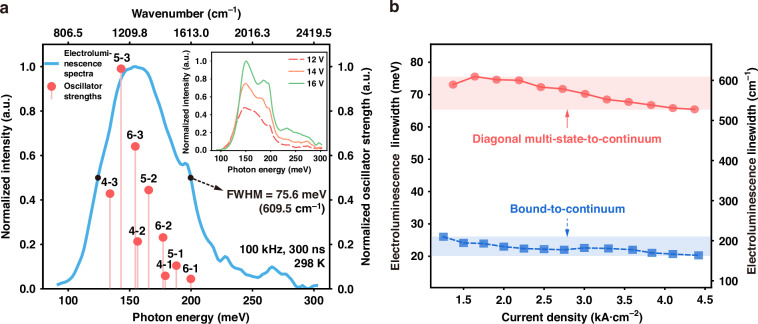
Characterization of electroluminescence (EL) and computational analysis of oscillator strengths for the diagonal multi-state-to-continuum (MTC) active region design. **a** Normalized oscillator strengths of optical transitions from the upper lasing levels (levels 4–6) to all lower levels (levels 1–3) are compared with experimentally measured intersubband electroluminescence (EL) spectra of the mesa device at 298 K under an applied bias of 11 V, with a pulse width of 300 ns and a repetition rate of 100 kHz. The inset shows the experimentally measured EL spectra of the same device recorded at various applied voltages under identical temperature and operation conditions. **b** The full width at half maximum of the spectra for the diagonal multi-state-to-continuum (MTC) device as well as for the bound-to-continuum (BTC) devices, respectively, as a function of the current density

After material characterization, mesa structures of different diameters were fabricated to investigate the spectral properties. The EL spectra of the device were measured under pulsed operation (100 kHz, 300 ns) at bias voltages ranging from 10.2 to 18 V. The spectrum recorded at 11 V is presented in Fig. 3a, while the inset displays the EL spectra observed between 12 and 16 V. The corresponding FWHM of the EL spectra, together with the spectra taken from a BTC design, are shown in Fig. 3b. A broad FWHM of ~70 meV is observed across the bias range, with a maximum value of 75.6 meV, corresponding to 609.5 cm^−1^ at a voltage of 11 V. This value is significantly broader than those reported for DAU/SS^18^ and BTC^17^ designs, and even the state-of-art DAU/MS designs^38^ in the same wavelength region. A detailed comparison of the critical performances of different designs is summarized in Supplementary Table S1.

The calculated oscillator strengths of the transitions from multiple upper states to the lower states are presented in Fig. 3a and the values are shown in Supplementary Table S2. These results show that the dominant oscillator strengths reasonably match the EL intensity spectra. Even compared with the continuum-to-continuum (CTC) design at a close wavelength^39^, the MTC shows more evenly distributed oscillator strength, thereby leading to an even broader EL spectrum, as shown in Fig. S1 of the Supplementary Material. We noticed that a few peaks at high energy (~190 meV and ~250 meV) are observed, especially for the higher voltage (i.e. rollover voltage of 16 V). We postulated that the peak at ~190 meV likely originates from the thermal effects (see Fig. S2 of the Supplementary Material), while the peak at ~250 meV is due to the injection into the parasitic energy level 7^17^. As shown in Fig. 3b, compared to the MTC design, the FWHM of the EL spectra for the BTC design remains below 30 meV and decreases with increasing bias. The detailed EL spectra of the BTC structure are shown in Fig. S4b. This clearly demonstrates that the MTC design can provide a significantly broader gain bandwidth.

**Fig. 4:**
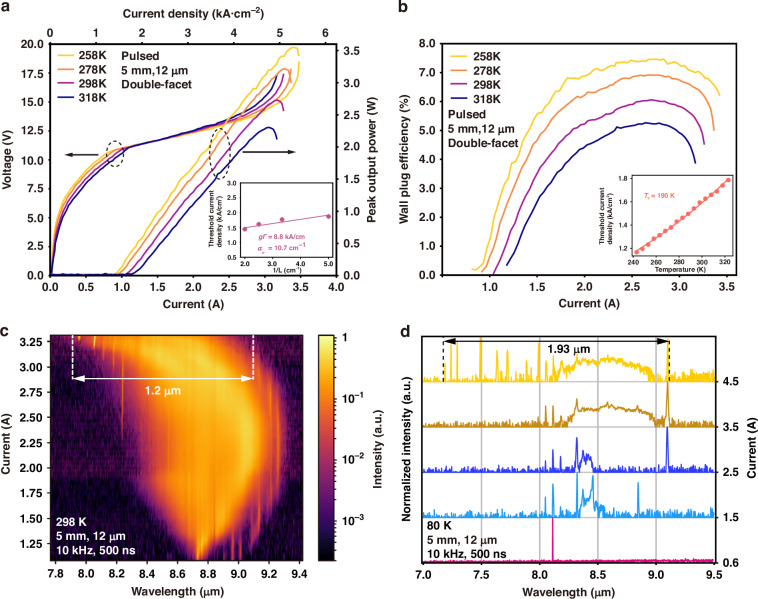
Lasing characteristics of the diagonal multi-state-to-continuum (MTC) device. **a** Light output power versus current (L–I) and voltage versus current (V–I) curves of a laser device under pulsed operation (500 ns, 10 kHz) at different temperatures ranging from 258 K to 318 K. The cavity length is 5 mm, and the ridge width is 12 μm. The inset shows the pulsed threshold current densities as a function of the varying cavity lengths. **b** The wall-plug efficiency as a function of current at various temperatures. The inset illustrates the threshold current densities at different temperatures. **c** The lasing spectra of the diagonal multi-state-to-continuum device from threshold to rollover currents at 298 K. **d** The lasing spectra of the same device measured at 80 K

A comprehensive characterization of the fabricated devices was performed, as shown in Fig. 4. Figure 4a presents the peak output power-current-voltage (LIV) curves at various temperatures ranging from 258 K to 318 K. The laser device was driven by a 500 ns long current pulse with a repetition rate of 10 kHz. The maximum peak output power of 2.72 W was achieved at 298 K without considering the collection efficiency, with a threshold current density of 1.58 kA/cm^2^ and a slope efficiency of 1.3 W/A. The calculated differential resistance of the device is 1.5 Ω at 298 K. The WPEs of the device over the temperature range from 258 K to 298 K are illustrated in Fig. 4b. A maximum WPE of 6.1% was achieved at 298 K. The LIV characteristics of the BTC structure are depicted in Fig. S4a. Although the MTC design exhibits a higher current density than that of the BTC design (~1.0 kA/cm^2^), significant differences exist between the BTC and MTC devices regarding active region doping levels and laser wavelengths. Nevertheless, the relatively high current density is not an inherent limitation of the MTC design, as further discussed in Section 6 of the Supplementary Materials. As shown in the inset of Fig. 4a, a linear least-squares fit to the measured values of thresholds yields modal gain coefficients *gΓ* of 8.8 kA/cm, as well as total waveguide losses *α*_w_ of 10.7 cm^−1^, where *g* is the gain coefficient and *Γ* is the optical confinement factor. Though with a much broader spectrum, there is almost no gain coefficient deterioration compared with the BTC design and high-performance QCLs at similar wavelengths^40,41^. In the inset of Fig. 4b, threshold current densities were measured at temperatures from 240 K to 320 K at an interval of 10 K. The characteristic temperature (*T*_0_) can be deduced by using the usual exponential fit function *J*_th_ = *J*_0_exp(*T*/*T*_0_) where *T* is the heatsink temperature, and *J*_th_ is the threshold current density. The characteristic temperature *T*_0_ of the MTC design is determined to be ~190 K, as depicted in the inset of Fig. 4b, and this value is comparable with the high-performance broad-gain QCLs emitting at similar wavelengths^31^.

Figure 4c presents 2D spectrum maps corresponding to different currents at 298 K. Cross-sectional spectra corresponding to Fig. 4c are provided in Supplementary Material, Fig. S3b. The single-stack MTC-QCL encompasses a spectral range from 7.9 μm to 9.31 μm. At the rollover current density, it attains a maximum continuous spectral width of 1.2 μm, extending from 7.9 μm to 9.1 μm. As evident in Fig. 4c, in the vicinity of the threshold current, the spectral width is relatively narrow, and the lasing peak is predominantly located at 8.8 μm. As the current increases (prior to reaching 2.4 A), the spectrum gradually broadens, and the center wavelength gradually redshifts towards 9.05 μm. The center wavelength blueshifts to 8.48 μm with further increase in the current. We notice that when the current exceeds 3.13 A, strong discrete lasing peaks around 8 μm appear, as shown in Fig. 4c, the physical origin of which will be explained later. Owing to its extremely broad-gain bandwidth, the device shows a gap-free spectral width of 1.2 μm at room temperature (RT), representing the broadest emission spectrum achievable by a single-stack QCL operating at a close wavelength at RT. With a cavity free spectral range of ~0.33 cm^−1^, the laser supports ~505 longitudinal modes, yielding an estimated average peak power of 4.1 mW per mode. The shift of the spectrum center wavelength with the bias validates the diagonal nature of the proposed active region MTC design structure. The specific tuning behavior of the transition energy results from the combination of the Stark effect^42^ and the nature of multiple radiative transitions between the upper and lower energy states involved.

To investigate the low-temperature spectral width of the laser, the spectral characteristics of the device were also evaluated at 80 K. Near the threshold current, the lasing spectrum is very narrow, with the central peak at 8.1 μm, as illustrated in the first curve (pink) of Fig. 4d (current at 0.6 A). As the injection current increases, the spectrum gradually broadens, and the highest peak appears at 9.11 μm. Around the rollover current, the spectral width extends from 8.05 μm to 9.11 μm. The central peak of the lasing spectrum is primarily located at 8.32 μm, while the highest peak remained at 9.11 μm, as illustrated in the fourth curve (orange) of Fig. 4d (current at 3.5 A). As the current continues to increase, new lasing peaks emerge at 7.18 μm and 7.98 μm. During this process, the spectrum continuously broadens, with the highest peak shifting from 9.11 μm at 3.5 A to 7.49 μm at 4.5 A. At a current of 4.5 A (orange-yellow curve in Fig. 4d), due to the strong mode competition effect, a multitude of new lasing peaks appear, while the spectrum attains its maximum bandwidth. Specifically, at 80 K, the spectral coverage extends over a range of 1.93 μm, from 7.18 μm to 9.11 μm, representing the broadest spectrum achieved by a single-core QCL to date. It should be highlighted that the bandwidth of the proposed structure is almost the same as the previous design^43^, which consists of four different wavelength stacks. Given the intriguing presence of lasing peaks near 8 μm in the vicinity of the rollover current, as depicted in Fig. 4c, a series of experiments was conducted to rule out any possible spectral artifact arising from nonlinearities in the measurement setups under different testing conditions. Meanwhile, far-field measurements were also conducted to get a deeper understanding of the relationship between the lasing frequency and the emission pattern.

**Fig. 5:**
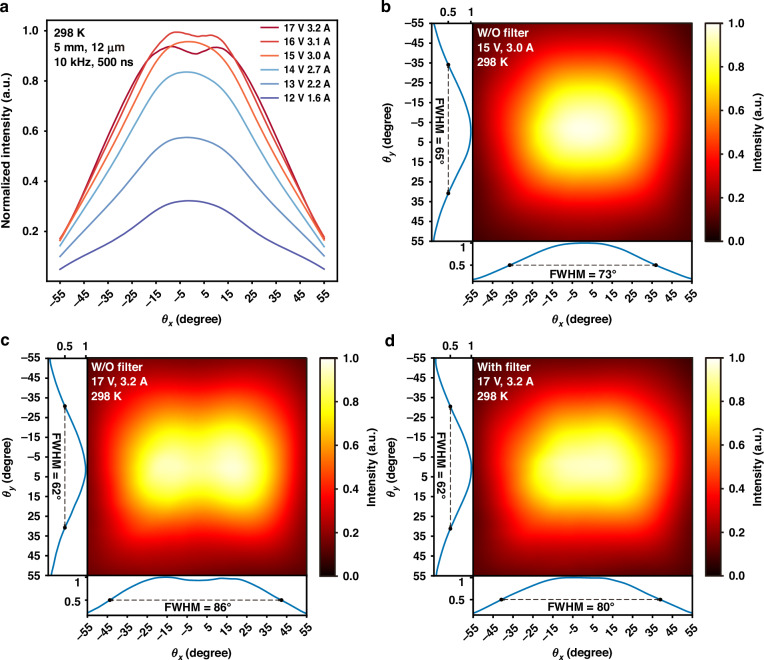
Far-field emission patterns of the diagonal multi-state-to-continuum (MTC) device with and without the filter. **a** One-dimensional far-field distribution in the horizontal direction at different currents in pulsed condition (10 kHz, 500 ns). **b** Two-dimensional (2D) far-field distribution at a current of 3.0 A. **c** 2D far-field distribution when the current is close to the rollover current (3.2 A). **d** 2D far-field distribution of the laser after passing through a filter (3.2 A)

**Fig. 6:**
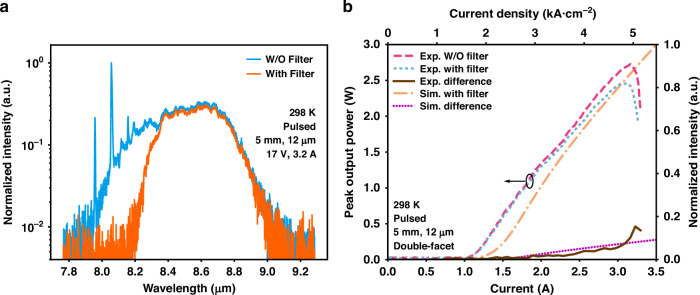
Optical power-current (L–I) characteristics and emission spectra with and without the filter, along with theoretical normalized power intensities of different modes. **a** Comparison of the spectra of the device with and without a long-pass filter. **b** Light output power-current-voltage (L–I–V) curves of the laser with and without filter, along with the theoretical normalized power intensities of the fundamental mode and the first high-order mode

First, the one-dimensional (1D) far-field patterns in the horizontal direction under different currents were measured, as shown in Fig. 5a. When the current is below 3.0 A, the far-field pattern shows only a single lobe, indicating fundamental transverse mode operation of the device. As the current ramping up to the rollover point, the far-field pattern displayed two weak lobes. Two-dimensional (2D) far-field measurements at currents of 3.0 A and 3.2 A are shown in Fig. 5b, c, respectively. To explore the relationship between the sharp lasing peaks at 8 μm and the far-field, a long-pass filter with a cutoff wavelength of 8.2 μm was placed in front of the laser, and the spectrum and far-field at 3.2 A were measured afterwards. As shown in Fig. 5d, after filtering, the far-field reverted to a single-lobe pattern. Therefore, the transition from a single-lobe to a two-lobe pattern effect is strongly associated with the emergence of strong lasing peaks at ~8 μm, as shown in Fig. 6a.

To investigate mode competition between the fundamental transverse mode and the high-order transverse mode in detail, the *L–I* curves of both with and without filters were measured, as shown by the blue and pink dashed curves in Fig. 6b. At low current, the laser power with and without the filter exhibited negligible difference. However, when the current increased to ~1.8 A, the laser power of the filtered laser became significantly lower than that of the unfiltered laser, indicating that the high-order mode began to compete with the fundamental mode. Above 1.8 A, the difference in light power, represented by the brown solid curve in Fig. 6b, between the unfiltered and the filtered curves increased progressively. This indicates that with increasing current, the high-order transverse mode gains more power in mode competition, a trend that culminated above the rollover current.

To numerically analyze the mode competition process between the two transverse modes, we employed the rate equation to model the variation in photon flux density of the fundamental transverse mode and the first high-order transverse mode as a function of the current, as described in ref. ^44^. As the current density shows significantly greater variation in the lateral direction than in the vertical direction, for simplicity, we assume the mode is uniform in the growth direction. Therefore, the mode competition can be treated as a 1D problem. In the simulation, the cross section of the active core is divided into M regions (M = 10) by M partitions along the lateral in-plane direction. The local electron sheet density *N*_3,*m*_ of the upper states, *N*_2,*m*_ of the lower states, and local photon flux densities *φ*_1_ and *φ*_2_ are calculated by a set of rate equations for the active region:

1.1 $$\frac{d{N}_{3,m}}{dt}=\frac{{J}_{inj,m}}{q}-\frac{{N}_{3,m}}{{\tau }_{3}}-{\sigma }_{32}({\varphi }_{1}{p}_{1,{\rm{no}}rm,m}+{\varphi }_{2}{p}_{2,{\rm{no}}rm,m})({N}_{3,m}-{N}_{2,m})$$

1.2 $$\frac{d{N}_{2,m}}{dt}=\frac{{N}_{3,m}}{{\tau }_{32}}+{\sigma }_{32}({\varphi }_{1}{p}_{1,norm,m}+{\varphi }_{2}{p}_{2,norm,m})({N}_{3,m}-{N}_{2,m})-\frac{{N}_{2,m}}{{\tau }_{2}}$$

1.3 $$\frac{d{\varphi }_{1}}{dt}=\frac{c}{n}{\varphi }_{1}\left\{\frac{{\varGamma }_{1}{\sigma }_{32}{\sum }_{m=1}^{M}[{p}_{1,norm,m}({N}_{3,m}-{N}_{2,m})]}{{L}_{p}{\sum }_{m=1}^{M}{p}_{1,norm,m}}-{\alpha }_{1}\right\}$$

1.4 $$\frac{d{\varphi }_{2}}{dt}=\frac{c}{n}{\varphi }_{2}\left\{\frac{{\varGamma }_{2}{\sigma }_{32}{\sum }_{m=1}^{M}[{p}_{2,norm,m}({N}_{3,m}-{N}_{2,m})]}{{L}_{p}{\sum }_{m=1}^{M}{p}_{2,norm,m}}-{\alpha }_{2}\right\}$$

where *J*_*inj,m*_ represents the local current density, *φ*_1_ and *φ*_2_ correspond the local photon flux densities in the center element (with *m* = *M*/2) of the fundamental transverse mode and the first high-order transverse mode, respectively. The normalized photon flux densities *p*_*1,norm,m*_ and *p*_2*,norm,m*_ are defined as *p*_*1*,norm,m_ = *φ*_1,m_/*φ*_1_ and *p*_2*,norm,m*_ = *φ*_2,m_/*φ*_2_, respectively. These normalized photon flux densities are obtained from the 2D optical mode simulation. The lifetimes of the energy level are estimated as τ_3_ = 1.45 ps and τ_2_ = 0.2 ps, with the nonradiative lifetime τ_32_ = 0.76 ps. The lifetimes are calculated by only considering the LO-phonon scattering times, following refs. ^16,44^. The thickness of one period of the active region is *L*_*p*_ = 44.8 nm, and the cross section of the optical transition is estimated to be *σ*_32_ = 1.07 × 10^−18^ m^2^. The optical losses for the fundamental mode and the first high-order mode are *α*_1_ = 10.7 cm^−1^ and *α*_2_ = 14.2 cm^−1^, respectively, and Γ_1_ ~ Γ_2_ ~ 0.6 are the mode confinement factors for both modes.

Based on Eq. (1.1)–(1.4), for a given injected current *I*, the lateral current distribution is calculated using a self-consistent, iterative numerical method (see section 7 in the Supplementary Materials). The calculated photon flux density (normalized power intensities) is presented in Fig. 6b. The orange dashed-dot (purple dotted) curve depicts the variation of the fundamental (first high-order) mode photon flux density with increasing current. From the results, it is evident that close to the threshold current, the photon flux density of the fundamental mode rises rapidly with the increase in current, and the first high-order mode starts to lase at approximately 1.6 A. Notably, the simulation results are in nice agreement with the experimental findings, thus verifying the transverse mode competition dynamics within the cavity. Interestingly, the “Exp. difference” curve in Fig. 6b, which exhibits a power increase when the current exceeds 3.1 A, is most likely attributed to pronounced longitudinal mode competition occurring at wavelengths above and below 8 μm (see Fig. S6 in Section 8 of the Supplementary Material).

## Discussion

In conclusion, to achieve a homogeneously broadband mid-infrared QCL, we have developed a diagonal MTC design utilizing multiple diagonal transitions to broaden the gain spectrum. A record 75.6 meV FWHM of EL is obtained at RT from the mesa device. Compared with the BTC devices fabricated in the same batch, the obtained EL spectrum remains over 2.6 times larger at the same current densities. Owing to the ultra-broadband nature of the active region, under pulse conditions, the measured emission spectrum shows a maximum width of 1.2 μm at RT, and the spectral bandwidth extends to 1.93 μm at 80 K. Despite the ultra-bandwidth, a peak power of 2.72 W, with a WPE of 6.1%, is still achieved, showing comparable performance with the state-of-art QCL at similar wavelengths^31^. Additionally, to explain the appearance of the strong spectral peaks around 8 μm, a transverse mode competition model based on the iterative rate-equation method has been formulated, showing a reasonable agreement with the experiments. Although the device currently operates only under pulsed conditions, the threshold and rollover current densities of our MTC devices closely match those reported for DAU structures in refs. ^19,45^ as well as for the three-phonon-resonance design in ref. ^41^. In addition, the characteristic temperature *T*_0_ is comparable to the values presented in ref. ^31^. It is worth noting that all of these high-performance lasers have demonstrated CW operation. By contrast, the current MTC device is uncoated, employs a non-buried-heterostructure configuration, and is epi-side up mounted. Given these structural differences and the high performance of the MTC design, we expect that implementing a buried-heterostructure design along with a high reflection coating will notably improve thermal conductivity, lower the threshold current density, and thereby enable CW operation. As for an even broader gain spectrum, as demonstrated in ref. ^46^ for BTC QCLs, two active regions emitting at distinct wavelengths can be monolithically stacked without fundamental limitations. The MTC design differs from BTC by the inclusion of additional transition energy levels, introducing no new physical constraints that would preclude a similar stacked configuration. Therefore, multi-wavelength stacking is, in principle, applicable to the MTC design, as it is to the BTC design.

We believe that the proposed MTC design represents an ideal candidate for a QCL-based mid-infrared frequency comb, whose bandwidth is limited by the available gain bandwidth of the active regions. Nowadays, the research on QCLs combs has emerged as one of the most captivating areas within the field, yielding significant advancements. For instance, the discoveries of dissipative Kerr soliton^47^ and quantum walk laser^48^ have been facilitated through the incorporation of innovative waveguide architectures. However, to achieve fully locked combs, QCLs with octave-spanning emission spectra are preferable to enable the *f-2f* locking scheme. With this consideration in mind, the stacking of MTC designs with different wavelengths emerges as a promising solution to this enduring challenge. Concurrently, the novel design also holds substantial potential for laser pulse compression^11,49^, when combined with meticulous group dispersion engineering and efficient RF injection.

## Materials and methods

### Growth

InAlAs/InGaAs/InP QCLs were grown on (100) n-InP substrates by MOCVD in a planetary reactor with H_2_ as the carrier gas and reactor pressure of 50 mbar. Trimethylaluminum (TMAl), trimethylgallium (TMGa), and trimethylindium (TMIn) were used for group III precursors, and phosphine and arsine as group V precursors. Si_2_H_6_ was used as the n-type dopant. The growth temperature was 670°C as measured by emissivity-corrected optical pyrometry. The InAlAs and InGaAs were grown with a single TMIn source. Growth rates for QCL active components (In_0.593_Ga_0.407_As and In_0.362_Al_0.638_As) were 0.1–0.5 nm/s, and no growth interruption was used between InAlAs and InGaAs interfaces. InP layers were grown at a higher rate of 0.3–0.8 nm/s. The V/III ratios were 90–150 for InAlAs and InGaAs, and 70–120 for InP. Epilayer structures were grown nominally strained to the (100) n-InP substrates, doped 2 × 10^18 ^cm^−3^.

### Fabrication

For laser performance characterization, the wafer was processed into a double trench configuration with a ridge width of 10−18 μm. The ridges were first defined and etched using an inductively coupled plasma-reactive ion etching reactor (ICP-RIE) with a Cl_2_/H_2_ plasma. After etching to form a trench with a depth of ~10 μm and a width of ~10 μm, a 500-nm-thick Si_3_N_4_ was deposited as an electrical isolation layer using plasma-enhanced chemical vapor deposition. The current injection windows were opened on top of the ridge by RIE. The Ti/Au metallization was evaporated as the top contact with E-beam evaporation. Finally, the sample was thinned down to around 150 μm, and the AuGe/Ni/Au metallization bottom contact was evaporated, followed by an annealing process performed at 290 °C for 40 s. The wafers were cleaved into 5 mm bars, and epi-up soldered on the copper heatsinks with indium solder for further characterization. For comparison, devices based on the BTC active region design at a slightly shorter wavelength, ~8.2 μm, were also fabricated using the same process in the same fabrication batch.

### Measurement

In the experiment, the laser was powered by pulse generators (AVTECH AVR-7B-B), mounted on a water-cooled platform, and positioned with the chip facet in close proximity to a calibrated thermopile detector (Ophir-3A). The temperature was meticulously monitored using a thermistor and precisely controlled via a thermoelectric cooler. The spectral characteristics of the device were characterized using a Fourier transform spectrometer (Bruker VERTEX 80 v) with a spectral resolution of ~0.07 cm^−1^. The far-field patterns were scanned by a two-axis goniometer far-field measurement setup.

## Supplementary information

Supplementary Information for “Ultra-broadband single-stack mid-infrared semiconductor lasers grown by MOCVD”

## Data Availability

The data presented in the manuscript are available from the corresponding author upon reasonable request.
