# Supplementary material for: Ultra-broadband single-stack mid-infrared semiconductor lasers grown by MOCVD
Source: Light Sci Appl. 2026 Apr 10;15:196. doi: 10.1038/s41377-026-02268-8 (PMC13068974; doi:10.1038/s41377-026-02268-8)
Supplement: Supplementary file 1 — Supplementary Information for “Ultra-broadband single-stack mid-infrared semiconductor lasers grown by MOCVD” [file 41377_2026_2268_MOESM1_ESM.docx]

**Supplementary Information for**

**“Ultra-broadband single-stack mid-infrared semiconductor lasers grown by MOCVD”**

**Peng Liu^1,2^, Lequan Zhang^1,2^, Yujin Wu^3^, Huanyu Lu^1,2^, Sicong Tian^4,2^, Bo Meng^1,2^*, Qi Jie Wang^3^, Cunzhu Tong^1,2^, Lijun Wang^1,2^**

1. State Key Laboratory of Luminescence Science and Technology, Changchun Institute of Optics, Fine Mechanics and Physics, Chinese Academy of Sciences, Changchun 130033, China

2. University of Chinese Academy of Sciences, Beijing 100049, China

3. School of Electrical and Electronic Engineering, Nanyang Technological University, Singapore 639798, Singapore

4. Bimberg Chinese-German Center for Green Photonics, Changchun Institute of Optics, Fine Mechanics, and Physics, Chinese Academy of Sciences, Changchun 130033, China

Corresponding author: [mengbo@ciomp.ac.cn](mailto:mengbo@ciomp.ac.cn)

Table of Contents

[**1.** **Differences between the MTC design and the CTC design** 3](#_Toc218073068)

[**2.** **Analysis of the multiple peaks in EL spectra** 3](#_Toc218073069)

[**3.** **Reproducibility of broadband lasers** 4](#_Toc218073070)

[**4.** **Bound-to-continuum active region structure** 5](#_Toc218073071)

[**5.** **Comparison of key performance metrics for single-stack QCLs operating at 8–10 μm** ………………………………………………………………………………………………………………………………………6](#_Toc218073072)

[**6.** **Analysis of the threshold current difference between BTC and MTC devices** 7](#_Toc218073073)

[**7.** **Detailed numerical calculations based on an iterative method** 10](#_Toc218073074)

[**8.** **An analysis of mode competition near the rollover point** 11](#_Toc218073075)

1. **Differences between the MTC design and the CTC design**

Compared with the continuum-to-continuum (CTC) design proposed in Refs. [1] and [2], the key difference between the MTC and CTC designs lies in the distribution of oscillator strengths. As shown in Fig. S1, our calculations reveal that the oscillator strengths in the MTC design are more evenly distributed across multiple transitions, whereas in the CTC design, only three transitions exhibit significantly higher oscillator strengths than the others. Consequently, the gain bandwidth of the MTC design is considerably broader than that of the CTC design.


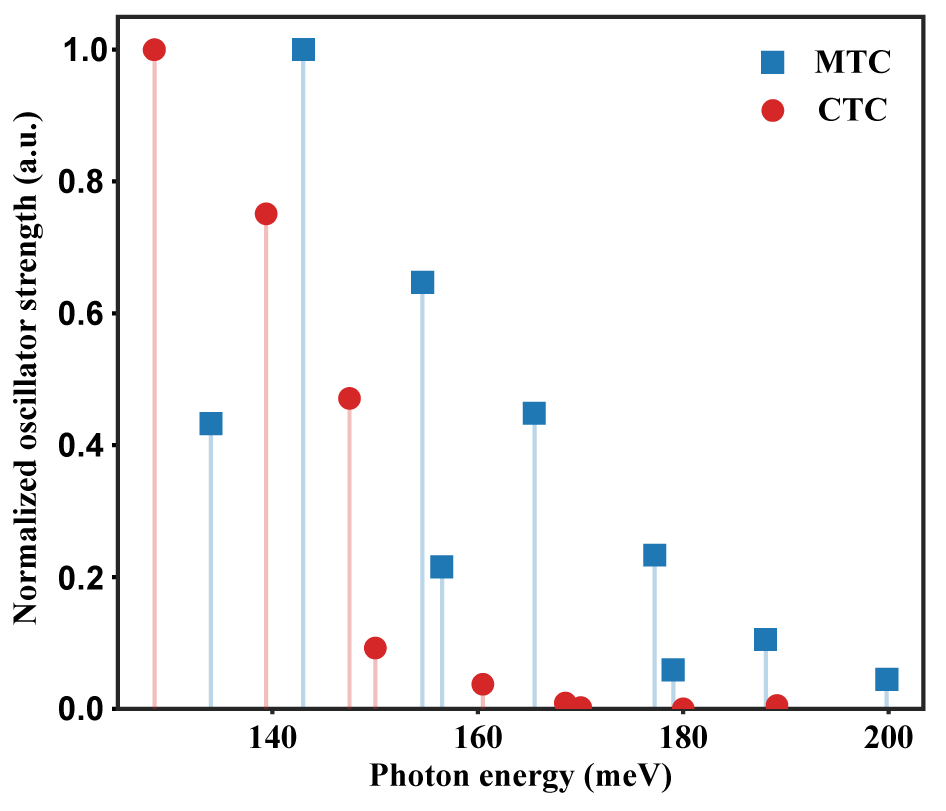


**Fig. S1.** Oscillator strengths of the main optical transitions for the diagonal multi-state-to-continuum (MTC) and continuum-to-continuum (CTC) design.

1. **Analysis of the multiple peaks in EL spectra**

In the MTC design, numerous transitions with small energy spacings exist—evidenced by the calculated oscillator strengths—making precise identification of individual peak positions challenging. Based on experimental observations, we propose that multiple additional peaks likely originate from thermal effects and parasitic high-energy states transitions. To investigate the thermal effect, EL devices of varying sizes were fabricated, and comparative measurements were performed. The results demonstrate a significant reduction in the number of spurious peaks at ~190 meV in smaller devices, as illustrated in Fig. S2. This suggests that thermal effects may contribute to the emergence of multiple peaks in the EL spectrum. The peak at ~250 meV could be attributed to the parasitic level transition. Injection into the parasitic high level from the injector level has been observed in different designs^3^, exhibiting similar EL behavior to the MTC design.


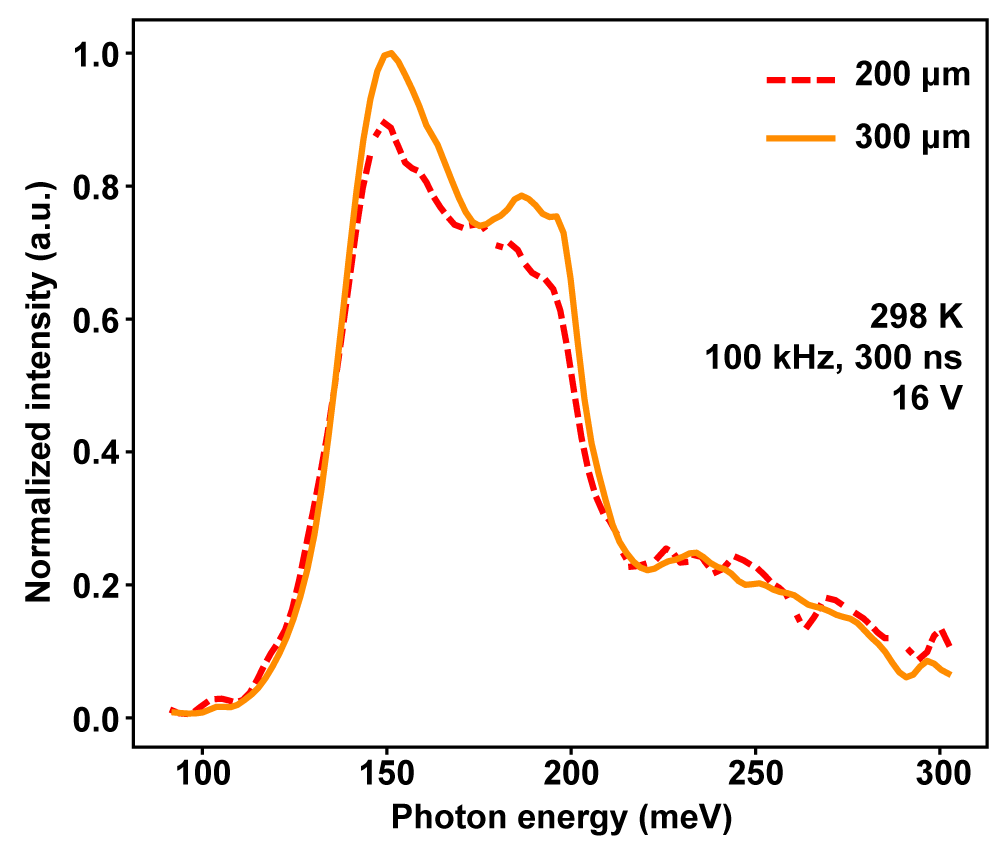


**Fig. S2.** Electroluminescence (EL) spectra of mesa-structured devices with different sizes measured under identical applied voltage conditions.

1. **Reproducibility of broadband lasers**

Multiple devices of identical size were subjected to pulsed operation testing at a frequency of 10 kHz and a pulse width of 500 ns. Fig. S3a and Fig. S3c present the lasing spectra of two identically sized MTC QCLs, measured from threshold current to rollover current at 298 K. Fig. S3b and Fig. S3d display the corresponding cross-sectional spectral profiles, providing a detailed view of the spectral evolution under increasing injection levels. The spectral widths measured across these devices were consistently around 1.2 μm. The uniformity of the spectral widths obtained from numerous devices strongly demonstrates the stability and reproducibility of the active region design.


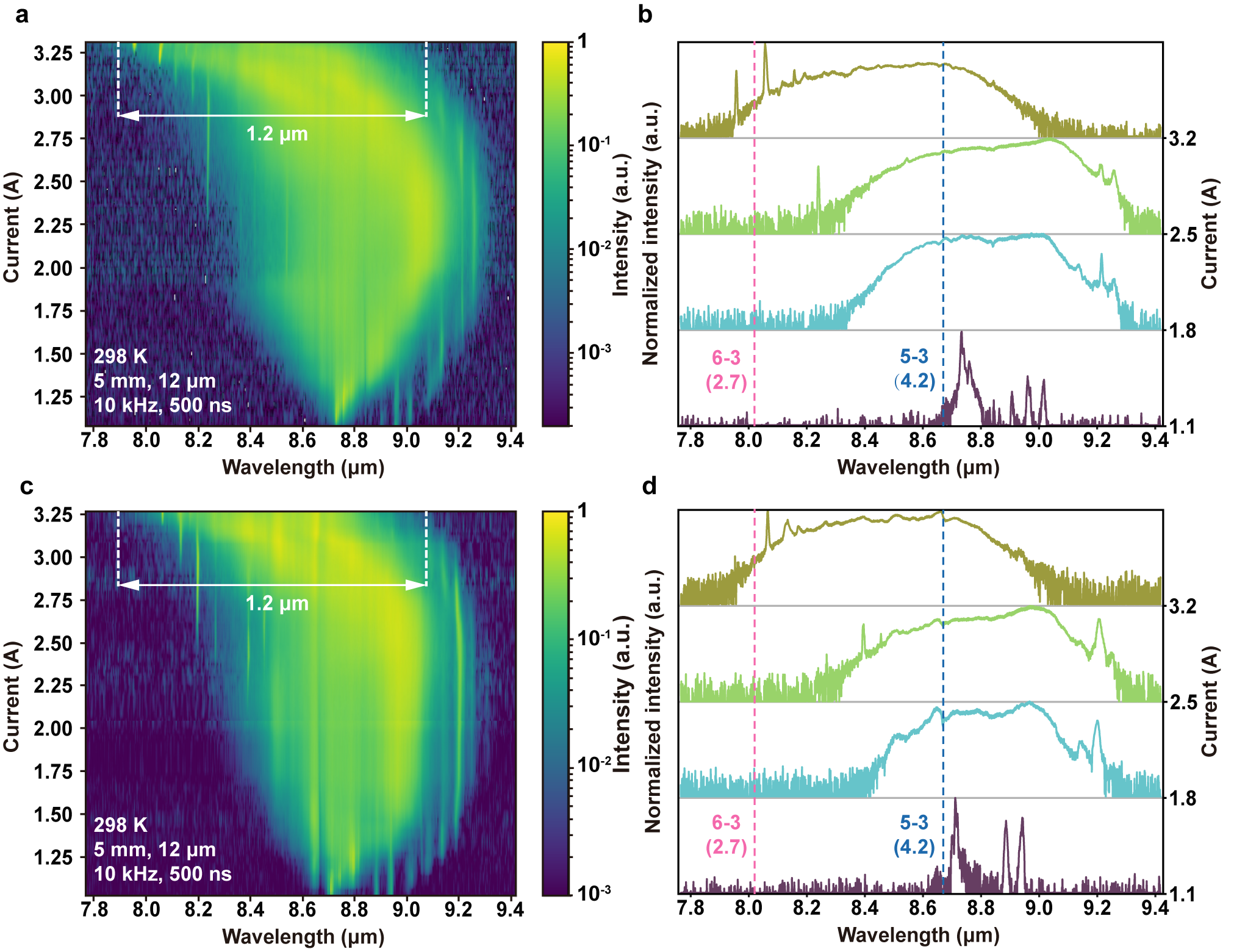


**Fig. S3.** (a) The lasing spectra of the diagonal multi-state-to-continuum (MTC) device from threshold to rollover currents at 298 K. (b) The cross-sectional spectral profiles derived from (a), along with the dominant oscillator strengths corresponding to optical transitions from the upper lasing levels to lower levels. The value in parentheses denotes the oscillator strength associated with the corresponding transition. (c) The lasing spectra of another MTC device from threshold to rollover currents under the same temperature. (d) The cross-sectional spectral profiles derived from (c), along with the dominant oscillator strengths corresponding to optical transitions from the upper lasing levels to lower levels. The experimentally measured emission spectra for all devices were obtained at 298 K with a pulse width of 500 ns and a repetition rate of 10 kHz.

1. **Bound-to-continuum active region structure**

The active region of the device consists of 35 stages of lattice-matched In_0.532_Ga_0.468_As quantum wells and In_0.521_Al_0.479_As barriers. The entire QCL structure was epitaxially grown on an *n-*doped InP substrate with a doping concentration of 2×10^18^ cm^−3^. The epitaxial layer sequence starting with a 0.5 μm thick *n-*doped (5×10^16^ cm^-3^) InP layer is as follows: a 3.0 μm thick *n-*doped (2×10^16^ cm^−3^) InP cladding layer, 35 periods InGaAs-InAlAs active structure (*n-*doped, 7.5×10^16^ cm^−3^), and a top waveguide cladding layer [consisting of a 3.3 μm thick *n-*doped (2×10^16^ cm^−3^) InP layer, a 0.4 μm thick *n-*doped (2.5×10^17^ cm^−3^) InP layer, a 0.3 μm thick *n-*doped (2.5×10^18^ cm^−3^) InP layer].

**
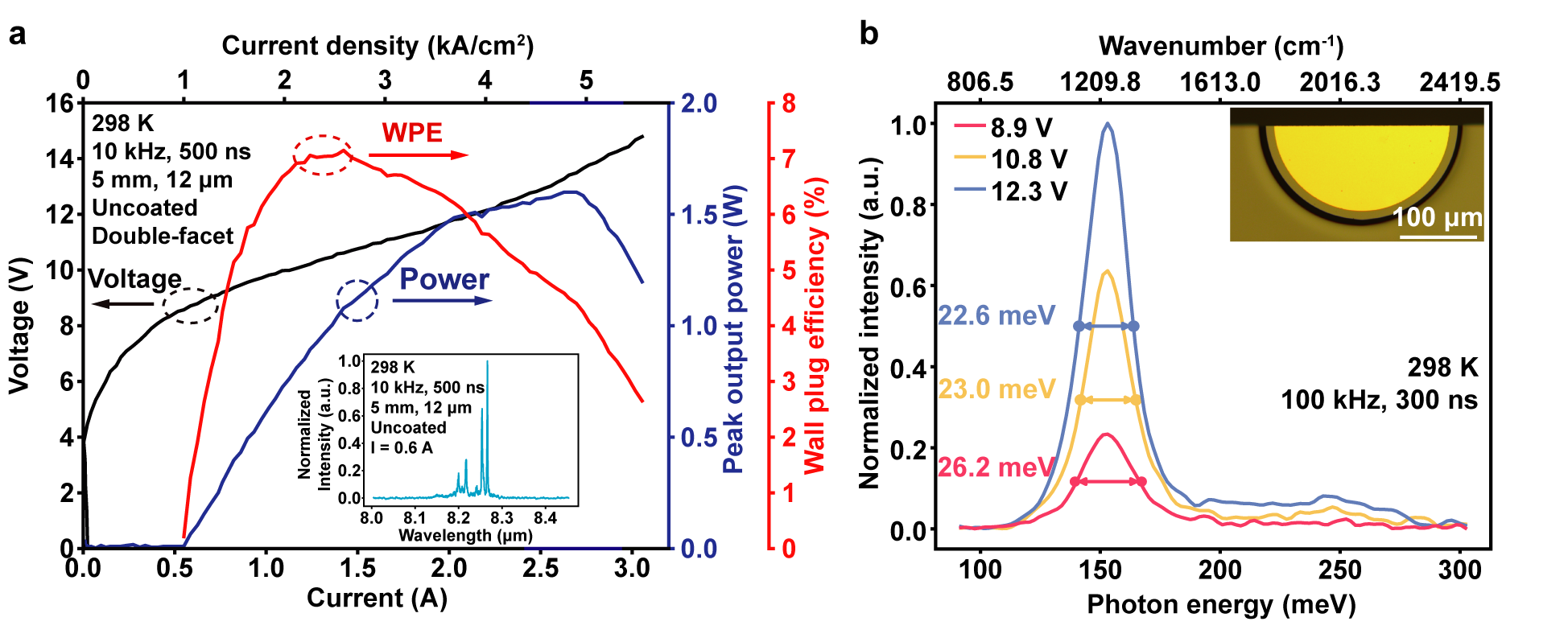
**

**Fig. S4.** (a) The light-current-voltage (*L-I-V*) characteristics of a 12 μm wide, 5 mm long device based on bound-to-continuum design at 298 K, with a pulse width of 500 ns and a repetition rate of 10 kHz. The inset in the lower right corner illustrates the spectral characteristics of the device near the threshold current (0.6 A). (b) Electroluminescence (EL) spectra of the mesa device for various biases. The inset is the microscope image of the mesa structure.

This wafer was also fabricated into a 5 mm long and 12 μm wide Fabry–Pérot (FP) QCL. The light-current-voltage (*L-I-V*) curves of this device are shown in Fig. S4a. Similarly, the electroluminescence (EL) spectra of the device under varying voltages were measured, with the full width at half maximum (FWHM) of the emission peaks remaining below 30 meV (see Fig. S4b).

1. **Comparison of key performance metrics for single-stack QCLs operating at 8–10 μm**

**Table S1.** Comparison of key performance metrics for single-stack QCLs operating at 8–10 μm.

| Year | Active region design*^a^* | FWHM of the EL spectrum at RT (cm^-1^)*^b^* | Center wavelength of the EL spectrum (cm^-1^) | Laser spectral range at RT (μm)*^c^* | The maximum RT peak power (W)*^d^* | *T*_0_ (K)*^e^* | *η* (W/A) *^f^* | WPE (%)*^g^* | Ref. |
| --- | --- | --- | --- | --- | --- | --- | --- | --- | --- |
| 2004 | BTC | 297 | ~1008 | ~0.8 | 0.51 | — | ~0.5 | — | [4] |
| 2008 | BTC | ~299 | ~1209 | — | ~0.60 | — | ~0.9 | ~4.6 | [3] |
| 2010 | CTB | ~375 | ~1100 | — | ~2.00 | 170 | 1.0 | ~2.9 | [5] |
| 2010 | DAU | 330 | ~1209 | ~0.5 | 0.61 | 306 | 1.0 | ~4.8 | [6] |
| 2011 | CTB | 200 | 1230 | — | — | — | — | — | [7] |
| 2011 | DAU/MS | 500 | ~1049 | ~0.5 | 0.93 | 510 | 1.0 | ~2.9 | [8] |
| 2012 | AC-DAU | — | — | ~0.3 | >0.13 | 525 | — | ~3.2 | [9] |
| 2015 | CTC | 444 | ~952 | 0.75 | 1.16 | — | 1.0 | ~3.1 | [1] |
| 2022 | DAU/MS | 466 | ~1150 | — | 1.55 | 228 | ~1.6 | 8.1 | [10] |
| 2023 | DT | ~260 | ~1180 | — | 4.25 | 217 | ~1.9 | 10.4 | [11] |
| 2025 | MTC | 610 | 1204 | 1.2 | 2.72 | 190 | 1.3 | 6.1 | This work |

*^a^*Notes: “SPS” is short period superlattice; “AC” is anti-crossed; “M” is modified; “DT” is diagonal-transition design.

*^b^*“RT” is room temperature; “FWHM” is full width at half maximum.

*^c^*“RT” is room temperature.

*^d^*The value applies to HR-coated QCLs or to both facets of uncoated QCLs; “RT” is room temperature.

*^e^*“*T*_0_” is characteristic temperature; The value applies to pulsed QCLs.

*^f^* “*η*” is slope efficiency.

*^g^*“WPE” is wall-plug efficiency.

1. **Analysis of the threshold current difference between BTC and MTC devices**

A comparison between Fig. S4a and Fig. 4a reveals that the MTC device exhibits a higher threshold current than the BTC device. However, it is important to note that despite the higher current density observed in the MTC design, there are notable differences between the BTC and MTC devices in terms of their active region doping levels and laser wavelengths. The MTC design features an active region doping level of 1.5×10^17^ cm^-3^ and operates at a laser wavelength of 8.8 µm, whereas the BTC design has a doping level of 0.75×10^17^ cm^-3^ and a wavelength of 8.2 µm. These differences in doping levels and wavelengths inherently result in increased free carrier absorption and intersubband absorption losses for the MTC design.

The free carrier absorption loss, derived from the complex propagation constant *k*, is given by *α*_w0_ = 2Im{*k*}, under the assumption of negligible losses in the active region. The free carrier absorption losses for the BTC and MTC devices, obtained from finite-element method (FEM) simulations, are 2.5 cm^-1^ and 2.9 cm^-1^, respectively. The overlap factor Г was also computed using FEM simulation, yielding values of 0.63 for the BTC device and 0.59 for the MTC device. The intersubband absorption can be calculated by the following expression^12^:

where the oscillator strength reads:

with Boltzmann’s constant *k_B_*, refractive index *n*_AR_, vaccum speed of light *c*, length of one period *L_p_*, half-width at half-maximum (HWHM) of the linewidth broadening *γ_ij_*, chemical potential *μ*, electron mass *m*_0_, dipole matrix element *z_n,n’_*, and the energy levels *E_n_* and *E_n’_* of initial *n* and final state *n*’. The calculated oscillator strengths for optical transitions from the upper lasing levels (levels 4–6) to all lower levels (levels 1–3) in the MTC design are summarized in Table S2.

**Table S2.** Oscillator strengths for optical transitions from the upper lasing levels (levels 4–6) to all lower levels (levels 1–3) in the MTC design.

| Oscillation strength | *f*_6,3_ | *f* _6,2_ | *f* _6,1_ | *f* _5,3_ | *f* _5,2_ | *f* _5,1_ | *f* _4,3_ | *f* _4,2_ | *f* _4,1_ |
| --- | --- | --- | --- | --- | --- | --- | --- | --- | --- |
| Value | 2.7 | 1.0 | 0.2 | 4.2 | 1.9 | 0.4 | 1.8 | 0.9 | 0.3 |

The calculation shows that the intersubband absorption losses Γα_ISB_ of the BTC and MTC devices are 3.4 cm^-1^ and 5.0 cm^-1^, respectively. Finally, the total waveguide losses can be expressed as follows:

resulting in total waveguide losses of 5.9 cm^-1^ and 7.9 cm^-1^ for the BTC and MTC designs, respectively. The calculated waveguide losses are in a reasonable agreement with the experimental values of 6.2 cm^-1^ and 10.7 cm^-1^ obtained using the 1/L method measurement shown in Fig. S5. A linear least-squares fit to the measured threshold current densities yields modal gain coefficients *gΓ* of 8.8 kA/cm for the MTC and 10.1 kA/cm for the BTC. Compared with Refs. [13, 14], the MTC device exhibits high modal gain that is on par with those of the state-of-art devices at similar wavelengths. Thus, it can be concluded that the relatively high total waveguide loss is not a fundamental limitation of the MTC design.


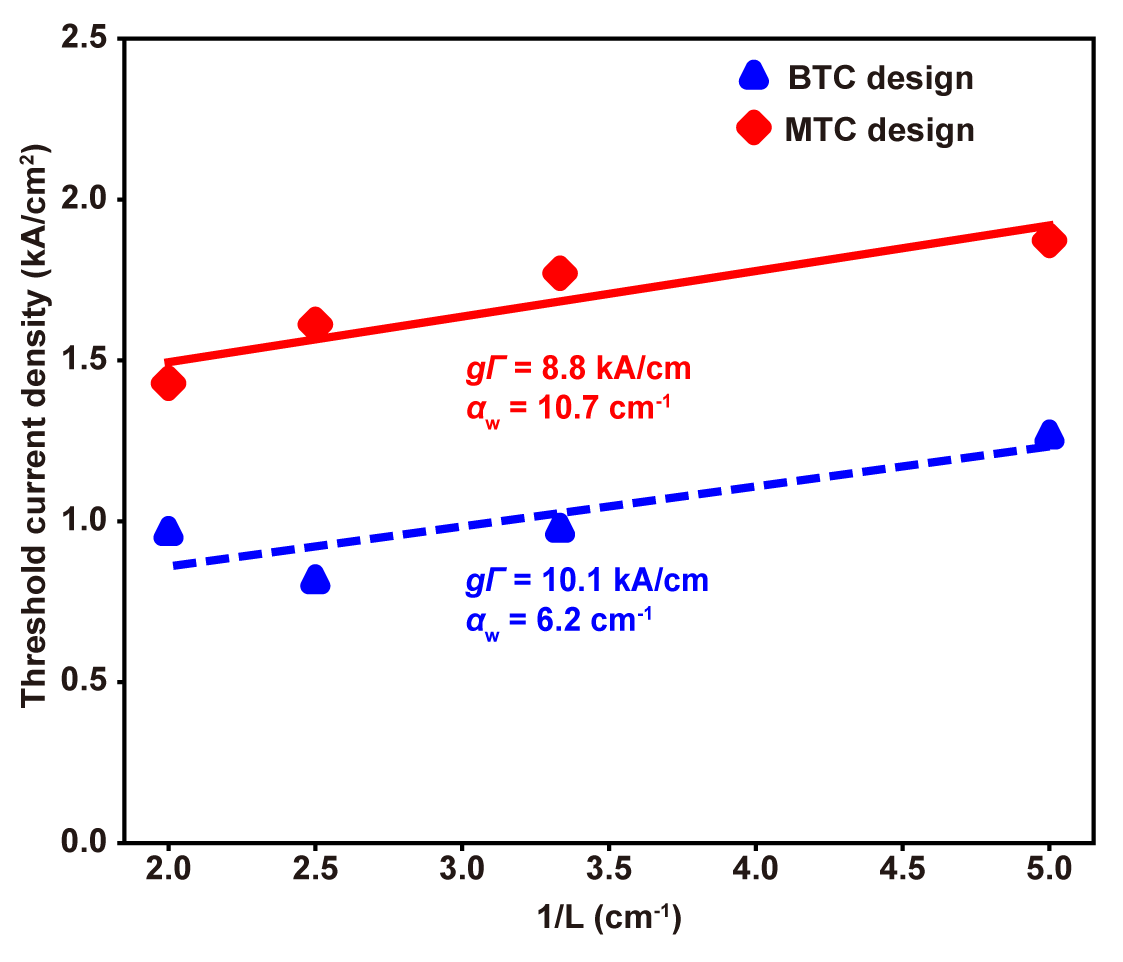


**Fig. S5.** Pulsed threshold current densities of MTC and BTC devices as a function of the cavity length.

1. **Detailed numerical calculations based on an iterative method**

In the *k*-th iteration step of the iteration, the rate equations can be expressed as:

where *dt* is the discretization time step, and the total effective lifetime τeff *3,m,k* of the upper level is obtained as,

Then the calculation proceeds to the (*k*+1)-th iteration step. Since the local current density is determined by the sheet electron density of the upper state *N*_3_*_,m,k_* and its total effective lifetime τeff 3*,m,k*, the injected current *I* is distributed among them according to *N*_3_*_,m,k_*/τeff 3*,m,k*. Thus, the local current density *J_m,k+_*_1_ for the (*k*+1)-th step is estimated based on the results from the *k-*th step by

$$J_{m,k+1}=\frac{N_{3,m,k}/\tau_{3,m,k}^{\text{eff}}}{\sum_{m=1}^{M} N_{3,m,k}/\tau_{3,m,k}^{\text{eff}}}\frac{I}{WL}M$$

where *W* represents the ridge width, and *L* denotes the cavity length. First, *J_m,k+_*_1_ is computed according to Eq. (6). Subsequently, similar to the *k*-th step, *N*_3_*_,m,k+_*_1_ and *τ*eff 3*,m,k+*1 are calculated using Eqs. (4) and (5), respectively. The obtained results will then be utilized to calculate *J_m,k+_*_2_ in the (*k+*2)-th step, again following Eq. (6). The iteration is terminated once the calculations converge to the steady state for the photon flux density *ϕ* as well as the electron densities *N*_3_*_,m_* and *N*_2_*_,m_*.

1. **An analysis of mode competition near the rollover point**

In Fig. 6b, the “Exp. Difference” curve exhibits a power increase when the injected current exceeds 3.1 A. At this current level, the QCL approaches the rollover point, where intense mode competition arises within the device, as demonstrated in Fig. S6. At a current of 3 A, no lasing mode is observed close to 8 μm. As the current increases, a spectral peak emerges slightly above 8 μm, as indicated by the red curve in Fig. S6 (*I* = 3.2 A). With further current increase, another peak appears slightly below 8 μm and subsequently dominates the emission spectrum, as shown by the blue curve in Fig. S6 (*I* = 3.3 A).

Therefore, the power rise observed in the "Exp. Difference" curve beyond 3.1 A is most likely attributed to pronounced longitudinal mode competition occurring at wavelengths both above and below 8 μm.


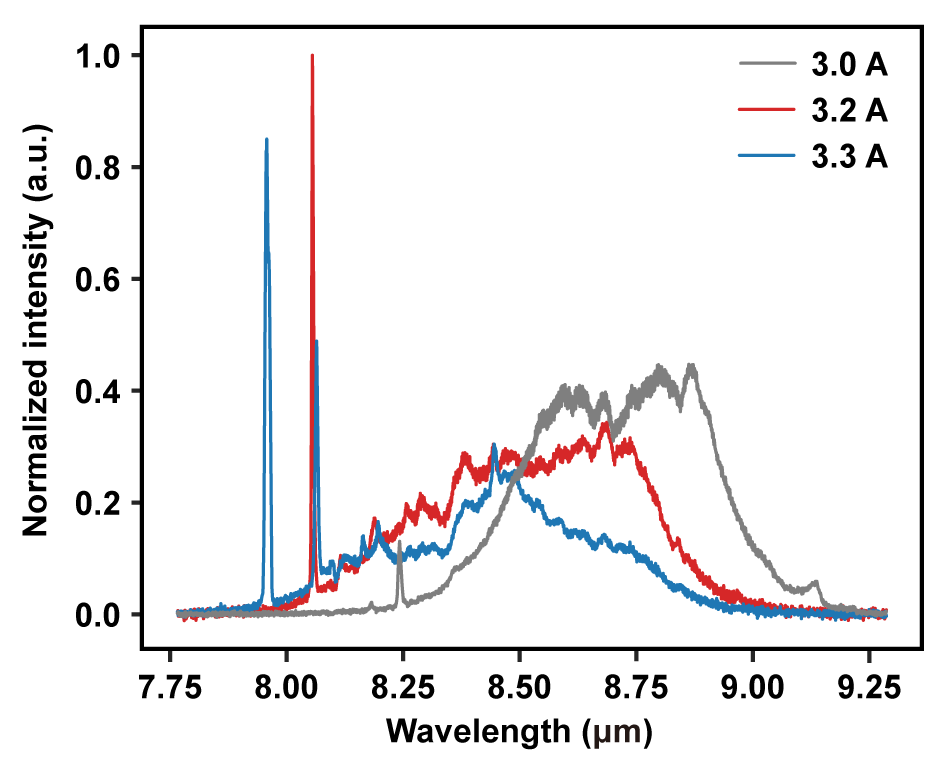


**Fig. S6.** Comparison of the spectra in the vicinity of 3.1 A.

**References**

1. Meng, B. *et al.* Broadband quantum cascade laser at wavelength λ∼10 μm based on continuum-to-continuum design. Proc. 2015 Conference on Lasers and Electro-Optics (CLEO), 2015, pp. 1-2.

2. Meng, B. *et al.* Broadly continuously tunable slot waveguide quantum cascade lasers based on a continuum-to-continuum active region design. *Appl. Phys. Lett.* **107**, 111110 (2015).

3. Wittmann, A. *et al.* Intersubband linewidths in quantum cascade laser designs. *Appl. Phys. Lett.* **93**, 141103 (2008).

4. Maulini, R. *et al.* Broadband tuning of external cavity bound-to-continuum quantum-cascade lasers. *Appl. Phys. Lett.* **84**, 1659-1661 (2004).

5. Yao, Y. *et al.* Broadband quantum cascade laser gain medium based on a “continuum-to-bound” active region design. *Appl. Phys. Lett.* **96**, 211106 (2010).

6. Fujita, K. *et al.* High-performance, homogeneous broad-gain quantum cascade lasers based on dual-upper-state design. *Appl. Phys. Lett.* **96**, 241107 (2010).

7. Mujagić, E. *et al.* Two-dimensional broadband distributed-feedback quantum cascade laser arrays. *Appl. Phys. Lett.* **98**, 141101 (2011).

8. Fujita, K. *et al.* Broad-gain (Δλ/λ0~0.4), temperature-insensitive (T0~510K) quantum cascade lasers. *Opt. Express* **19**, 2694-2701 (2011).

9. Fujita, K. *et al.* Extremely temperature-insensitive continuous-wave quantum cascade lasers. *Appl. Phys. Lett.* **101**, 181111 (2012).

10. Sun, Y. *et al.* High-performance quantum cascade lasers at λ ∼ 9 µm grown by MOCVD. *Opt. Express* **30**, 37272-37280 (2022).

11. Niu, S. *et al.* High power, broad tuning quantum cascade laser at λ ∼ 8.9 µm. *Opt. Express* **31**, 41252-41258 (2023).

12. Wittmann, A. *et al.* High-Performance Bound-to-Continuum Quantum-Cascade Lasers for Broad-Gain Applications. *IEEE J. Quantum Electron.* **44**, 36-40 (2008).

13. Zhou, W. *et al.* High performance monolithic, broadly tunable mid-infrared quantum cascade lasers. *Optica* **4** (2017).

14. Wang, Q. J. *et al.* High performance quantum cascade lasers based on three-phonon-resonance design. *Appl. Phys. Lett.* **94**, 011103 (2009).
